# COMMENTARY ON: PRELIMINARY STUDY: EFFICACY OF FOCUSED SHOCKWAVE THERAPY IN PATIENTS WITH MODERATE-TO-SEVERE CARPAL TUNNEL SYNDROME

**DOI:** 10.2340/jrm.v56.40610

**Published:** 2024-06-11

**Authors:** Juhi SINGH, Digvijay SHARMA, Adarsh Kumar SRIVASTAV

**Affiliations:** Department of Physiotherapy, School of Health Sciences, Chhatrapati Shahu Ji Maharaj University, India

We recently reviewed the article by Vongvachvasin et al. (1), with keen interest. We wish to express our appreciation to the authors for their valuable contribution in assessing the effectiveness of focused extracorporeal shockwave therapy for managing this condition. The authors’ thorough examination revealed a notable reduction in T-BCTQ symptoms and function scores across both treatment groups, particularly in favour of focused extracorporeal shockwave therapy throughout the study duration. Additionally, the observed discrepancies in distal sensory and motor latency between the groups at the 3-week mark from baseline offer meaningful insights into the therapeutic benefits of this intervention. This study not only enhances our comprehension of carpal tunnel syndrome treatment but also highlights the potential of focused shockwave therapy as a promising therapeutic approach.

In recent years, the management of moderate-to-severe carpal tunnel syndrome (CTS) has witnessed the emergence of focused shockwave therapy as a promising non-invasive intervention (2). This innovative approach offers a valuable alternative for patients who may be reluctant to undergo surgery or have contraindications for more invasive treatments. Studies have shown that focused shockwave therapy can lead to notable improvements in pain relief, functional status, and quality of life in individuals with CTS (2). By targeting the underlying pathology of CTS, such as nerve compression and tissue inflammation, focused shockwave therapy holds the potential to alleviate symptoms and improve hand function without the need for surgical intervention. Additionally, its role as an adjunctive therapy alongside conservative treatments underscores its versatility in optimizing CTS management. However, further research is needed to evaluate its long-term efficacy, safety profile, and cost-effectiveness compared with traditional interventions. Nonetheless, the clinical relevance of focused shockwave therapy in the treatment paradigm of moderate-to-severe CTS is increasingly recognized, offering new avenues for improving patient outcomes and quality of life.

The absence of blinding of participants in a study poses a significant risk of performance bias. Performance bias occurs when participants’ awareness of their treatment allocation influences their behaviour or responses, potentially leading to biased study outcomes. Furthermore, unblinded participants may inadvertently influence outcome assessors, compromising the objectivity of outcome assessments. Blinding is crucial in research as it helps minimize the risk of bias, enhances the internal validity of the study, and ensures that observed effects are more likely attributable to the intervention itself rather than other factors (3).

The lack of specificity in the inclusion criteria of the article, as compared with the registered trial, is concerning. Failure to precisely define inclusion criteria can compromise the validity of study results and hinder the generalization of evidence. Additionally, the rationale behind selecting a specific gender for inclusion should be provided, as this could potentially impact the interpretation and applicability of the findings. To improve the specificity of the evidence generated, it is recommended to incorporate the duration of symptoms along with the baseline assessment of patients, such as the Visual Analog Scale (VAS) score of pain. This addition would enhance the precision of the inclusion criteria and provide a valuable context for understanding treatment effects (4).

Additionally, there is a lack of clarity concerning how the sample size was calculated, as there are inconsistencies between the stated method and the actual computation. The study references a specific formula for sample size determination, {(*n* = [(Zα/2 + Zβ)^2^ × {2(σ)^2^}]/ (μ1 - μ2)^2^}, but the resulting sample size does not match the values obtained using G*Power software (5). Specifically, when G*Power 3.1.9.7 was employed with a *t*-test family, an alpha (error probability) of 0.05, a power (1-beta error probability) of 0.95, and an effect size of 0.5, the minimum required sample size was calculated to be 184. However, this number does not align with the reported number of recruited patients in the article. This discrepancy raises questions concerning the accuracy and reliability of the reported sample size determination process.

To comprehensively interpret the study’s findings, it is essential to delve into the effect size, calculated at 0.56 through the indirect method (6). This metric offers critical insights into the practical significance of the intervention’s impact on outcomes. A value of 0.56 indicates a moderate effect size, suggesting that the intervention may have clinically meaningful effects on the studied pathology. Moreover, the disparities noted in sample size, participant recruitment, and calculation methodologies compared with the reference article underscore the necessity for post hoc analysis. By conducting such analysis, we can meticulously scrutinize the data in light of these differences, enabling a more thorough understanding of the study’s results. Post hoc analysis may reveal additional patterns or associations that were not apparent initially, providing valuable insights into the study’s findings. Additionally, leveraging G*Power software 3.1.9.7 to determine a statistical power of 0.746 bolsters our confidence in the robustness of the intervention’s effects within the chosen pathology. A statistical power of 0.746 indicates a high likelihood of detecting true effects if they exist, further supporting the validity of the study’s conclusions. Collectively, these steps solidify the statistical underpinning of the study, enriching the validity and depth of its conclusions, and providing a more comprehensive understanding of the intervention’s efficacy in the context of the studied pathology.

In summary, while the preliminary findings of the study are promising, it is imperative to recognize the importance of robust methodology and transparent reporting in clinical research. Addressing methodological limitations and ensuring adherence to best practices in study design, conduct, and analysis are essential steps toward advancing evidence-based practice in the management of carpal tunnel syndrome. By fostering a culture of rigorous scientific inquiry and continuous improvement, we can enhance the quality of research in this field and ultimately optimize patient care. As such, future studies should aim to build on these findings, incorporating methodological refinements and larger sample sizes to further elucidate the efficacy and safety of focused shockwave therapy for carpal tunnel syndrome.
