# Supplementary material for: COMMENTARY ON: PRELIMINARY STUDY: EFFICACY OF FOCUSED SHOCKWAVE THERAPY IN PATIENTS WITH MODERATE-TO-SEVERE CARPAL TUNNEL SYNDROME
Source: J Rehabil Med. 2024 Jun 11;56:40610. doi: 10.2340/jrm.v56.40610 (PMC11196983; doi:10.2340/jrm.v56.40610)
Supplement: Related texts

# REPLY TO “COMMENTARY ON: PRELIMINARY STUDY: EFFICACY OF FOCUSED SHOCKWAVE THERAPY IN PATIENTS WITH MODERATE-TO-SEVERE CARPAL TUNNEL SYNDROME”

**Authors:** Pimpisa VONGVACHVASIN, Thitiporn PHAKDEPIBOON, Waree CHIRA-ADISAI, Punpetch SIRIRATNA

**Affiliations:** Department of Rehabilitation Medicine, Faculty of Medicine Ramathibodi Hospital, Mahidol University, 270 RamaVI road, Ratchathewi district, Bangkok 10400 Thailand

We express our gratitude for the opportunity to respond to the critical review of our recently published article (1). Although we acknowledge that not all methodological details desired by the reviewer were included in our paper, this does not alter the main conclusions of our study. After carefully reading this critical review, we disagree with its conclusion regarding the robustness of the methodology. Specific responses to major critiques of the review are outlined below.

Regarding study blinding, we agree that blinding should be employed whenever possible in clinical research as it can reduce the risk of performance bias and enhance the overall quality of the research. However, it was challenging to implement blinding in our study, such as use of a sham extracorporeal shockwave therapy (ESWT) instrument. Theoretically, to produce such blinding, a shockwave device that is capable of generating sound (so the patients would think they are being treated) without applying any energy to the patient, i.e., disconnecting the shockwave generator, would have to be employed. Unfortunately, a particular instrument like that intended for research purposes is currently unavailable in Thailand. Another alternative option for sham ESWT was to apply an extremely low dose of focused ESWT, for example, at a magnitude of 0.01 mJ/mm^2^. However, there is currently no consensus that even such a minimal dosage would not potentially provide some therapeutic effects. A further option to consider was to use an air-chambered polyethylene foil and the method described in a study by Gerdesmeyer et al. (7). Nevertheless, we were not aware of this option at that time. For these reasons, we did not administer the sham treatment to the control group.

For the sample size calculation, we acknowledge the limitation of a small sample size and agree that larger sample sizes almost always increase the confidence of and ability to generalize the results. However, in actual clinical settings, key factors including funding, time, resources, and numbers of participants available are often limited. In order to optimize our study when considering these factors, we calculated the minimum feasible sample size that could be conducted in our particular setting. In this study, we used the clinical superiority formula for continuous data (8) with a power of 80% (9) and the standard deviation from the study by Vahdatpour et al. (10). The figure of 0.5 in our study is the minimal clinically important difference for the functional status scale of the Boston Carpal Tunnel Syndrome Questionnaire based on a study by Leite et al. (11). This resulted in the total of 24 participants being required. We were fully aware of this small sample size, thus we termed our study a preliminary study. Clearly, further studies with larger sample sizes need be conducted to provide confirmation of these preliminary findings.

To summarize, we acknowledge the limitations as mentioned above. Nevertheless, we believe that the findings from this study are still beneficial to the community as they provide important initial evidence regarding the potential for ESWT in moderate-to-severe CTS. We agree that further studies with larger sample sizes are needed to extend the conclusions drawn from this study and to expand the knowledge and evidence for clinical application of ESWT in CTS.

## References

[1] Vongvachvasin P, Phakdepiboon T, Chira-Adisai W, Siriratna P. Efficacy of focused shockwave therapy in patients with moderate-to-severe carpal tunnel syndrome: a preliminary study. J Rehabil Med 2024; 56: jrm13411.38332536 10.2340/jrm.v56.13411PMC10865893

[2] Xie Y, Zhang C, Liang B, Wang J, Wang L, Wan T, et al. Effects of shock wave therapy in patients with carpal tunnel syndrome: a systematic review and meta-analysis. Disabil Rehabil 2022; 44: 177–188.32419508 10.1080/09638288.2020.1762769

[3] Schulz KF, Altman DG, Moher D. CONSORT 2010 statement: updated guidelines for reporting parallel group randomised trials. J Pharmacol Pharmacother 2010; 1: 100–107.21350618 10.4103/0976-500X.72352PMC3043330

[4] Raissi GR, Ghazaei F, Forogh B, Madani SP, Daghaghzadeh A, Ahadi T. The effectiveness of radial extracorporeal shock waves for treatment of carpal tunnel syndrome: a randomized clinical trial. Ultrasound Med Biol 2017; 43: 453–460.27814933 10.1016/j.ultrasmedbio.2016.08.022

[5] Sakpal TV. Sample size estimation in clinical trial. Perspect Clin Res 2010; 1: 67–69.21829786 PMC3148614

[6] Berben L, Sereika SM, Engberg S. Effect size estimation: methods and examples. Int J Nurs Stud 2012; 49: 1039–1047.22377339 10.1016/j.ijnurstu.2012.01.015

[7] Gerdesmeyer L, Wagenpfeil S, Haake M, Maier M, Loew M, Wörtler K, et al. Extracorporeal shock wave therapy for the treatment of chronic calcifying tendonitis of the rotator cuff: a randomized controlled trial. JAMA 2003; 290: 2573–2580.14625334 10.1001/jama.290.19.2573

[8] Zhong B. How to calculate sample size in randomized controlled trial? J Thorac Dis 2009; 1: 51–54.22263004 PMC3256489

[9] Eng J. Sample size estimation: how many individuals should be studied? Radiology 2003; 227: 309–313.12732691 10.1148/radiol.2272012051

[10] Vahdatpour B, Kiyani A, Dehghan F. Effect of extracorporeal shock wave therapy on the treatment of patients with carpal tunnel syndrome. Adv Biomed Res 2016; 5: 120.27563630 10.4103/2277-9175.186983PMC4976534

[11] Leite JC, Jerosch-Herold C, Song F. A systematic review of the psychometric properties of the Boston Carpal Tunnel Questionnaire. BMC Musculoskelet Disord 2006; 7: 78.17054773 10.1186/1471-2474-7-78PMC1624826
